# Caregiver’s Self-Confidence in Food Resource Management Is Associated with Lower Risk of Household Food Insecurity among SNAP-Ed-Eligible Head Start Families

**DOI:** 10.3390/nu12082304

**Published:** 2020-07-31

**Authors:** Lamis Jomaa, Muzi Na, Sally G. Eagleton, Marwa Diab-El-Harake, Jennifer S. Savage

**Affiliations:** 1 Department of Nutrition and Food Sciences, Faculty of Agricultural and Food Sciences, American University of Beirut, Riad El-Solh, P.O. Box 11-0236, Beirut 1107-2020, Lebanon; lj18@aub.edu.lb (L.J.); md106@aub.edu.lb (M.D.-E.-H.); 2 Department of Nutritional Sciences, Penn State College of Health and Human Development, 108C Chandlee Laboratory, University Park, PA 16802, USA; muzi.na@psu.edu (M.N.); sge5@psu.edu (S.G.E.); 3 Center for Childhood Obesity Research, Penn State College of Health and Human Development, University Park, PA 16802, USA

**Keywords:** food resource management, food insecurity, self-confidence, nutrition education, financial practices, SNAP-Ed, Head Start, young children

## Abstract

Food resource management (FRM) behaviors are key components within nutrition education programs designed to help food insecure households maximize their food dollars. However, little is known about the association between FRM self-confidence and financial practices with household food insecurity (HFI) among families with young children. Using a sample of SNAP-Ed-eligible Head Start families, this study examined associations between FRM self-confidence, FRM behaviors and financial practices by HFI. A needs assessment survey was conducted with caregivers of Head Start children (*n* = 365). HFI was measured using the US Household Food Security Survey Module. Chi-square and logistic regression analyses were conducted to examine if FRM self-confidence, FRM behaviors, and financial practices differed by HFI. Participants with high FRM self-confidence had lower odds of HFI (OR = 0.54, 95%CI: 0.33, 0.87), yet FRM behaviors, financial practices, and HFI were not related after adjusting for covariates. All FRM self-confidence questions significantly differed by HFI, whereas only one of six FRM behaviors and two of three financial practices differed by HFI (all *p*-values < 0.05). Promoting caregivers’ self-confidence in FRM skills within nutrition education programs may be explored as a potential strategy to assist low-income households to stretch their food dollars in an attempt to address HFI.

## 1. Introduction

Household food insecurity (HFI), defined as “the inability to provide enough food for a healthy and active lifestyle for all household members [1]”, remains a serious social and public health problem in the US [2]. Food insecurity is especially prevalent among low-income families with children. In 2018, 13.9% of American households with children were food insecure, and the prevalence of HFI reached 14.3% among households with children 6 years of age or younger [3]. Food insecurity is associated with a range of negative health outcomes among infants and young children, including poor physical health, increased risk of infections, micronutrient deficiencies [4,5], suboptimal sleep quality [6], adverse behavioral, mental, and academic behaviors [5,7,8], as well as obesity and other chronic conditions during childhood and later in life [7,9].

Federal assistance programs that provide monetary benefits along with nutrition education to low-income households have been shown to alleviate HFI [10]. These nutrition education programs, such as the Supplemental Nutrition Assistance Program Education (SNAP-Ed) and the Expanded Food and Nutrition Education Program (EFNEP), provide participants with trainings on how to maximize the use of their food dollars while providing healthy foods to their families and children [11]. An integral component of these nutrition education programs is to teach individuals how to acquire food resource management (FRM) skills and behaviors defined as “the handling of all foods and the resources that may be used to acquire foods by an individual or family [12].” In addition, FRM trainings cover topics such as meal planning, shopping strategies, food selection, budgeting, food preparation, and cooking strategies to maximize nutrition under resource constraints [12]. Previous studies indicate that integrating FRM within nutrition education (e.g., food preparation tips, healthful food selection, and budgeting) improves the food security status of low-income households [10,13], including those with children [14,15].

Although food assistance programs, such as SNAP and SNAP-Ed, focus on behavioral change in FRM, less emphasis has been placed on assessing participants’ self-efficacy and confidence in their FRM skills. Few studies, to date, have reported how nutrition education interventions targeting self-efficacy and confidence in FRM can improve food security [15,16]. Perceived self-efficacy represents a key construct in behavioral change theories, as it refers to an “individual’s confidence in their ability to plan and follow through with a series of actions that will result in desired outcomes or achievements” [17]. Research studies examining the effect of self-efficacy on behavioral change related to nutrition, exercise, and weight loss [18], as well as the prevention of chronic diseases [19], have demonstrated the pivotal role that self-efficacy plays in improving health. Knowing that families experiencing food insecurity may face various challenges affecting their confidence in managing their budgets to maintain food sufficiency [20,21], it is integral to further examine the association between FRM self-confidence and HFI [16].

Food insecurity is linked to income [1]; however, food insecurity is not the outcome of income alone. Instead, it is influenced by a myriad of other demographic, environmental, and financial factors [22,23]. To further examine the determinants of HFI, a growing body of literature has been exploring the association between financial management skills and food insecurity [22,24]. It was previously suggested that good financial management practices may safeguard certain households from food insecurity, whereas those with less effective financial skills may be at increased risk of food insecurity [22,25]. To our knowledge, the associations between FRM, financial practices, and HFI have not been adequately explored in the literature, particularly among households with young children. To address this research gap, the present study aimed to first examine the associations between FRM self-confidence and FRM behaviors by HFI status using a sample of SNAP-Ed-eligible Head Start families. A secondary objective of the study was to explore the association between financial practices of caregivers and HFI status in the study sample. Head Start is a federally-funded program that serves just over 900,000 low-income preschool children in the US to optimize their health and nutrition. The Head Start program also provides balanced snacks and meals to children through the Child and Adult Care Food Program [26]. Although previous studies have shown that Head Start programs can help alleviate HFI and improve nutrition outcomes of children [27,28], none, to our knowledge, have examined the potential associations between caregiver’s FRM self-confidence and behaviors by HFI. We hypothesized that (1) caregivers with higher self-confidence and better FRM skills would have lower risk of being food insecure; and (2) caregivers with good financial practices would report lower levels of food insecurity.

## 2. Materials and Methods

### 2.1. Sampling and Recruitment

Caregiver-child dyads in the present study were recruited from Head Start preschool classrooms in four rural counties in central Pennsylvania. Data used in the present study were drawn from a needs assessment survey that was designed to characterize the home environments of low-income families with young children and to better inform future nutrition education programming for the Head Start participants. The survey was distributed through classrooms to 1297 Head Start families. If parents had more than one child enrolled in Head Start, they were instructed to complete the survey for their oldest child enrolled in the program. Of the 1297 distributed surveys, 379 (30%) were returned in the mail. Caregivers received a $25 gift card for their participation. Data collection spanned May 2017 to May 2018. Among nine families, a survey was completed for two children in the home, thus we excluded the survey for the younger of the two children. Four children were excluded because they were outside the age range of Head Start eligibility, resulting in a final study sample of 365. For the purpose of the present study, a minimum sample size of 134 participants was required to test for the associations between our main variables of interest (FRM behavior, FRM self-confidence, and HFI) at 80% power and with 95% confidence interval. The sample size calculations were done using data from previous studies that examined similar associations [10,16]. Informed consent was obtained from subjects prior to their participation in the study. The study was conducted in accordance with the Declaration of Helsinki, and the protocol was approved by the Institutional Review Board at the Pennsylvania State University (00007467).

### 2.2. Caregiver and Household Characteristics

The survey included questions related to the caregiver characteristics, such as age and sex, ethnicity, education, employment, and marital status. As for household characteristics, questions included child’s age, number of children in the household, number of people supported by household income, participation in assistance programs in the past 12 months (e.g., Special Supplemental Nutrition Program for Women, Infants, and Children (WIC) and Supplemental Nutrition Assistance Program (SNAP)), and household income. Household income was missing in seventy-four of 365 households (20.2%). Missing income was imputed based on WIC and SNAP status, parent education, marital status, and employment using PROC MI in SAS software (SAS Institute Inc., Cary, NC, USA).

### 2.3. Household Food Insecurity Status

Household food insecurity (HFI) experienced during the previous 12 months was measured using the 18-item US Household Food Security Survey Module [1]. The food security status of households was determined by the number of food-insecure conditions and behaviors the household reports. Households were classified as ‘food secure’ if participants responded affirmatively to two or fewer items on the 18-item scale and as ‘food insecure’ if the affirmative responses were on three or more items, such as “cutting the size of meals or skipping meals because there wasn’t enough money for food during the past 12 months” or “losing weight because there wasn’t enough money for food”.

### 2.4. Food Resource Management (FRM) Self-Confidence and Behaviors

FRM self-confidence and FRM behaviors of caregivers were assessed in the present study using two sets of questions derived from the SNAP-Ed evaluation framework guide and toolkit [11]. These questions were previously used and validated in other studies assessing the impact of nutrition education programs targeting low-income adults, including SNAP-Ed, Cooking Matters, and Expanded Food and Nutrition Education Program (EFNEP), on participants’ FRM skills [10,14,29] and confidence [16,29].

The caregivers’ self-confidence in FRM abilities (in the past 12 months) was assessed in the present study using five questions. Three questions assessed caregiver confidence to “choose the best-priced form of fruits and vegetables”, “buy healthy foods on a budget”, and “cook healthy foods on a budget”; and two questions were related to caregiver’s confidence in their ability to “make a shopping list and stick to it” and “compare prices of similar foods to find the best value”. Responses for these questions were measured using a 4-point Likert scale that ranged from 1 (*not very confident*) to 4 (*very confident*). An average FRM self-confidence score was calculated for each participant based on their responses to the five questions, and a binary score was later created for FRM self-confidence to classify participants into two groups (low/high): participants with scores less than the median were categorized as “low” FRM self-confidence, whereas participants with scores greater than or equal to the median score were categorized as “high” FRM self-confidence. A high FRM self-confidence indicated a greater self-confidence in shopping, preparing foods, and managing food resources on a budget.

The FRM behaviors of participants in the present study were assessed using six questions from the SNAP-Ed evaluation framework and toolkit, asking how often do caregivers “plan meals before shopping”, “prepare shopping list”, “compare prices before buying”, “use grocery store flyers”, and “identify foods on sales or use coupons” [11]. A 5-point response scale (1 = *never*, 2 = *rarely*, 3 = *sometimes*, 4 = *usually*, 5 = *always*) was used for each of the FRM behavior items. An average FRM behavior score was first calculated, then a binary score was created to classify participants into two groups: participants with scores less than the median were categorized as “low” FRM behaviors, whereas participants with scores greater than or equal to the median score were categorized as “high” FRM behaviors. A high FRM behavior indicated better practices in meal planning, shopping with a grocery list, and comparing prices.

### 2.5. Financial Situation, Financial Practices, and Difficulties

To assess the financial situation, respondents were asked to describe their own financial situation with responses including: 1 = “*Very comfortable and secure*”, 2 = “*Very comfortable and secure*”, 3 = “*Occasionally have some difficulty making ends meet*”, 4 = “*Tough to make ends meet but keeping head above water*”, and 5 = “*In over your head*”. As for financial difficulties, these were evaluated based on 5 questions from the USDA national food study [30] to assess difficulties that individuals had in meeting their essential household expenses, such as mortgage or rent payments, utility bills, or important medical care during the past six months.

Financial practices of the caregivers were also assessed using 3 questions that were derived from the USDA national food study [30]. Caregivers were asked to report how frequently they adopted the following practices during the past 6 months: “review your bills for accuracy”, “pay your bills on time”, and “pay more than the “minimum payment due” on your credit card bills”. Response options ranged from 1 = never to 5 = always. An average financial practices score was calculated for each participant based on their responses to the five questions, and a binary score was later created (low/high): participants with scores less than the median were categorized as “low”, whereas participants with scores greater than or equal to the median score were categorized as “high”, referring to those with better financial practices.

### 2.6. Statistical Analyses

Descriptive statistics were reported in the present study as frequencies and proportions for categorical variables and as medians and interquartile ranges (IQR) for non-normal continuous variables. Chi-square tests and Mann-Whitney U tests were conducted to explore differences between categorical variables and non-normal continuous variables by HFI status (food secure vs. food insecure households), respectively. Simple and multiple logistic regression analyses were also conducted to examine the association between FRM self-confidence, FRM behaviors, and financial practices by HFI status. Variables included in the multiple logistic regression models were those found to have a significant bivariate relationship with HFI and were statistically significant in the simple logistic models (*p* < 0.05). Sensitivity analyses were also conducted to assess the validity of findings by: (1) adjusting for significant and non-significant sociodemographic variables as potential confounders in the logistic regression models, (2) running linear regression models with HFI and other variables of interest (FRM behavior, FRM self-confidence and financial practices) as continuous variables, and (3) running models using imputed and non-imputed income data. For the models with non-imputed income, we excluded subjects with missing income in the sensitivity analysis. Results from the logistic regression models were expressed as odds ratios with 95% confidence intervals. Statistical analyses were conducted using Stata/MP version 15.1 (StataCorp. College Station, TX, USA). A *p*-value of 0.05 was used to detect significance in all analyses used in the present study.

## 3. Results

### 3.1. Descriptive Characteristics of the Study Sample

The majority of caregivers in our study sample were females (96%), White non-Hispanic (98%), and completed high school education level or less (61%). The median age of caregivers was 30 (IQR = 9) years old. More than half of study participants were married or partnered (57%) and unemployed (54%). In addition, almost three quarters of participants were receiving SNAP benefits (75%) and WIC (70%). The median number of children in the household was 2, and the prevalence of HFI was 37% (see Table 1).

Caregiver and household characteristics of the study sample were also presented by HFI in Table 1. Participation in the SNAP/Food Stamps program was significantly greater among food insecure households compared to food secure ones (84% vs. 69%, *p* = 0.001), whereas participation in WIC was less common among food insecure households (64% vs. 74%, respectively, *p* = 0.041). No other significant associations were noted between HFI and demographic characteristics in the present study.

### 3.2. Food Resource Management and Household Food Insecurity

Table 2 presents FRM self-confidence and FRM behaviors of caregivers in the study sample and by HFI. Results showed that almost three-quarters of caregivers were *moderately* to *very confident* in choosing best priced food items, comparing food prices for best values, and cooking healthy food items on a budget. In addition, slightly greater than two-thirds of participants were *moderately* or *highly confident* in “buying health foods for their families on a budget” and “making a shopping list and sticking to it”. The proportion of participants reporting *usually* or *always* adopting FRM behaviors ranged between 31% and 79%. The less adopted FRM behaviors included “using grocery store flyers to plan meals” (31%), “planning of meals prior to grocery shopping” (57%), and “identifying foods on sale or using coupons to save money” (57%).

Significant differences were observed between food secure and food insecure households for all FRM self-confidence items (*p*-value < 0.05). More specifically, caregivers in food secure households were more likely to report being very confident in their abilities to “choose best priced fruits and vegetables” (42% vs. 27%), “buy healthy foods for their families” (41% vs. 21%), “cook healthy foods on a budget” (45% vs. 25%), “make a shopping list and stick to it” (43% vs. 26%), and “compare prices of similar foods when shopping to get the best value” (47% vs. 32%) when compared to their food insecure counterparts. On the other hand, only one item from the FRM behaviors was found to be significantly different between food secure and food insecure households in our study sample. A greater proportion of caregivers in food secure households reported that they always “use a shopping list when grocery shopping” as compared to their food insecure counterparts (49% vs. 33%, Table 2).

### 3.3. Financial Situation, Practices, and Difficulties and Household Food Insecurity

When caregivers were asked to describe the household’s financial situation, 37% of the total sample reported being “very comfortable and secure” or “able to make ends meet without much difficulty”, 34% “occasionally have some difficulty making ends meet”, and the remaining 29% reported it is “tough to make ends meet but keeping your head above water” or they are “in over their heads”. In terms of financial practices, the majority of caregivers in the study sample responded they *usually or always* “review bills for accuracy” (75%) and “pay bills on time” (79%), yet less than one-third of participants responded they “pay more than the “minimum payment due” on credit card bills” as frequently. With respect to financial difficulties, 39% of caregivers in our study reported going through a time “when they could not pay mortgage or rent, electricity or gas utilities, or important medical expenses”, and 44% reported going through periods when they “could not pay the full amount of gas, oil, or electricity bills” (Table 3).

In addition, Table 3 presents the financial situation, difficulties, and financial practices of caregivers in the study sample by HFI. Overall, food insecure households were more likely to report their financial situation as “occasionally have some difficulty making ends meet” (40% vs. 31%) or “tough to make ends meet but keeping head above water” compared to their food secure counterparts (45% vs. 14%). In terms of financial practices, a higher proportion of caregivers in food secure households reported they *always* “pay bills on time” (50% vs. 25%) and “pay more than the minimum payment due on credit card bills” (20% vs. 11%) compared to their food insecure counterparts. On the other hand, food insecure households were significantly more likely to report facing financial difficulties compared to food secure ones: “has there been a time when you could not pay your mortgage or rent, electricity or gas utilities, or important medical expenses?” (63% vs. 24%) and “has there been a time when you could not pay the full amount of gas, oil, or electricity bills” (64% vs. 32%), *p*-value < 0.001.

### 3.4. Food Resource Management, Financial Practices, and Household Food Insecurity

The associations between FRM self-confidence, FRM behaviors, and financial practices with HFI were also explored in the present study (Table 4). Results from the logistic regression analyses showed that caregivers with high FRM self-confidence had lower odds of HFI (OR = 0.54, 95% CI: 0.33, 0.87, *p* = 0.012), even after adjusting for financial practices and participation in food assistance programs (SNAP and WIC). Although the association between financial practices and HFI was significant in the simple regression analysis, this association lost its statistical significance in the adjusted model (OR = 0.77, 95%CI: 0.46, 1.3, *p* = 0.338). Results from the models remained robust after conducting sensitivity analyses and adjusting for significant and non-significant sociodemographic variables, including parent’s age, employment, household income (imputed and not imputed values), and participation in food assistance programs in the past 12 months (Supplemental tables—Tables S1 and S2).

## 4. Discussion

Food insecurity remains a social and public health problem for low-income families with young children in the US that has serious consequences on children’s overall health and wellbeing. To our knowledge, the present study is the first to examine the associations between FRM self-confidence, FRM behaviors, and financial practices by HFI status in a sample of low-income households with young children. Using a sample of SNAP-Ed-eligible Head Start families, our study findings showed that caregiver’s self-confidence in their FRM was associated with lower odds of HFI. Nevertheless, the associations between the FRM behaviors and financial practices of Head Start caregivers by HFI were not statistically significant in the adjusted models.

As hypothesized, caregivers with high FRM self-confidence had lower odds of HFI in the present study, even after adjusting for other correlates including FRM behaviors, financial practices and participation in other federal assistance programs. When individual FRM questions were explored, all FRM self-confidence questions were also found to significantly differ by HFI status. More specifically, caregivers in food secure households were more likely to report being “*very confident*” in their abilities to choose the best priced fruits and vegetables, compare prices of similar foods when shopping to get the best value, as well as buy and cook healthy foods for their families on a budget as compared to their food insecure counterparts. These results were in concordance with those reported earlier by Begley et al. (2019) showing that food secure participants, who were assessed at the enrollment stage of an adult food literacy program in Australia, reported being “*always confident*” about managing money for healthy food compared to food insecure participants (41.2% vs. 9%) and “*always confident*” in their ability to cook a variety of healthy meals (21.9% vs. 15.4%) [31]. Our findings were also consistent with a few studies conducted to date that highlight how greater self-efficacy in shopping and preparing healthy food, based on nutrition education programs targeting low income adults, has been associated with lower risk of food insecurity [16,29]. According to Martin et al. (2016), self-efficacy in managing food resources was found to be associated with a decrease in very low food security levels among food pantry clients participating in the *Freshplace* intervention. This was an 18-month innovative food pantry intervention that combined several strategies to boost the confidence of participants, such as motivational interviewing and serving food in client-choice format to increase their confidence in planning meals ahead of time, making a shopping list before going to the store, and making food money last all month [16]. Another study evaluating the impact of *Cooking Matters for Adults* nutrition education program showed significant improvements in the FRM skills and self-confidence in managing food resources of low-income households up to six months after the program completion. In addition, participants in the Cooking Matters intervention were worried less that food would run out before they could get money to buy more [29]. It is worth noting that these nutrition education programs were focused primarily on improving the self-efficacy of low-income adults as integral components for the uptake and maintenance of FRM skills to maximize the use of limited food dollars.

Although self-efficacy represents a key construct within theories of behavioral change and has been shown to be effective in promoting healthy behaviors for weight loss, exercise, and chronic disease management [32,33], only a few studies to date, as described earlier, have explored the association between self-confidence in FRM with food insecurity among low-income households [16,29]. To our knowledge, the present study is the first to examine these associations in low-income households, focusing primarily on those with young children. Our study findings suggest that increased confidence in resource management skills among caregivers may be associated with lower risk of HFI. These results may be promising for families with young children, who may have increased concerns about smart shopping, stretching their food dollars, as well as cooking tasty and low-cost food to feed their children [20,34]. Food insecure individuals may be also influenced by financial, social, and personal stressors that can further affect their confidence in their ability to shop, prepare, and plan a healthy meal on a limited budget [35,36]. Thus, federal assistance and nutrition education programs targeting families with young children, such as Head Start and SNAP-Ed, may need to give particular attention to strategies that can help improve the self-confidence and efficacy of caregivers in their resource management skills. These programs can also help participants in accessing community-level resources and in overcoming common misconceptions and barriers to enrolling in other federal assistance programs, including WIC [37].

Nevertheless, when exploring FRM behaviors, only one of the six behaviors of caregivers were shown to differ significantly by HFI in the present study. In addition, the association between FRM behaviors and HFI was not found to be statistically significant in the regression models. Contrary to our study findings, food secure families were previously observed to have overall better FRM skills, such as shopping for sales, researching for best prices on particular products, traveling to multiple stores, and planning meals around their limited budgets [35,38]. According to Begley et al. (2019), individuals who reported at the onset of a food literacy program a low frequency of adopting certain planning and food preparation behaviors, such as planning meals ahead of time and making a list before they shop, were significantly more likely to be food insecure than those who reported adopting more frequently these behaviors [31]. The limited differences in FRM behaviors by HFI, as observed in the present study, can be attributed in part to the overall low proportion of caregivers who reported planning their meals prior to grocery shopping, using grocery store flyers to plan their meals, or identifying foods on sale and using coupons to save money. Another reason could be differences in questions raised when assessing caregivers’ FRM confidence and behaviors in the present study. For example, questions relevant to buying and cooking healthy foods were only present in the FRM self-confidence questionnaire, whereas questions related to using shopping lists and planning meals prior to shopping were common among both scales. Caregivers participating in the present study may have also received family-centered services that cover topics related to child nutrition, growth, and development as part of the Head Start programs [39,40,41,42], which could have influenced their perceived confidence in providing healthy foods for their children. Nevertheless, confidence alone might be insufficient to alleviate HFI, and households with higher confidence may not be able to adopt adequate FRM behaviors when other environmental, financial, and personal barriers exist, such as limited availability and/or access to food stores with healthy and nutritious food, lack of kitchen appliances, as well as time and money constraints [31,38,43]. Poor physical and mental health can also affect the FRM skills and capabilities of food insecure individuals [38,44] and are worth further exploration when examining the association between resource management skills and HFI.

A growing body of evidence suggests that households facing economic hardships and with limited knowledge of basic financial concepts (i.e., financial literacy) are also more likely to experience food insecurity compared to those with higher financial management skills [22,25]. In line with former research, results from the present study showed significant differences in the financial situation, difficulties, and financial practices of caregivers by HFI status. Compared to caregivers from food insecure households, those from food secure households were more likely to report better financial situation and lower financial difficulties reflected through their ability to pay their mortgages or other basic expenses (such as rent, electricity, gas, and medical expenses). In addition, caregivers from food secure households were also more likely to report frequently adopting certain financial practices, such as paying bills on time and paying more than the “minimum payment due” on credit card bills. Nevertheless, the association between higher financial practices and HFI lost its statistical significance in the adjusted models. These results may be explained by the lower income levels of households enrolled in federal assistance programs, such as SNAP and WIC, who represent approximately three-quarters of the study sample, and who may be facing heightened financial hardships that could have attenuated the association between caregivers’ better financial management practices and their HFI status. Our study findings highlight the need to further explore the association between financial literacy (knowledge and capabilities) and HFI, particularly in low-income households with children. The latter group may be at increased risk of facing economic hardships, and thus may adopt risky coping strategies that can further increase their risk for HFI and its adverse health consequences [22,45].

### Strengths and Limitations

To our knowledge, this study is the first to explore the associations between FRM self-confidence, FRM behaviors and financial practices by HFI among a sample of low-income Head Start households with young children. Nevertheless, the present study has a number of limitations worth considering. First, the study is cross-sectional in nature, thus causality cannot be determined when exploring the associations between FRM self-confidence, FRM behaviors, and financial practices by HFI. The association between FRM self-confidence and food insecurity, as reported in the present study, may have been bidirectional in nature. Caregivers in food insecure households may have poor conditions that affect their self-confidence in their resource management skills as compared to food secure households; on the other hand, having higher self-confidence may also improve one’s capabilities to access and utilize food, which can influence their food security and feeling of self-sufficiency [31]. Another limitation of the present study is that data were self-reported, thus we cannot rule out response bias. Our study findings may also have limited representativeness with a moderate survey response rate (30%) and the study population limited to only four rural areas in central Pennsylvania. Thus, results cannot be generalizable and the external validity of our findings may be limited to certain low-income families. Albeit modest, the response rate in the present study was still similar to other surveys conducted with rural Head Start families in Colorado (28.5%) and Appalachian Ohio (42%) [46,47]. Future research considering more diverse and larger samples of Head Start families are still needed to further examine the associations explored in the present study.

## 5. Conclusions

Our study findings suggest that increased self-confidence in FRM among caregivers of young children is associated with lower odds of HFI among low-income Head Start families. Nutrition and health education programs, such as SNAP-Ed and WIC, that are designed to assist low-income households in alleviating their HFI status may need to give more emphasis to the self-efficacy and confidence of caregivers in stretching their food dollars and adopting adequate FRM skills. The strategies may help caregivers in offering healthy food and improve the food choices offered to their children. Caregivers can also play a pivotal role in structuring their children’s early experiences with food through child feeding practices, social modeling of healthy eating behaviors, and regulating the quality and quantity of food provided to the child [48,49,50]. Thus, future research should examine the extent to which nutrition education programs that focus on improving FRM self-confidence and behaviors can contribute (directly or indirectly) to the feeding practices of caregivers and, subsequently, to the diet quality and nutrition outcomes of young children in low-income households. It is also important to further investigate the role that financial literacy and practices of caregivers can play in improving the food security of low-income households.

## Figures and Tables

**Table 1:** Descriptive characteristics of a sample of low-income Head Start families with preschool- aged children from four rural counties in central Pennsylvania, USA, by household food insecurity status, (*n* = 365) ^1,2,3^.

	Total Sample (*n* = 365)	Food Secure (*n* = 229)	Food Insecure ^4^ (*n* = 136)	*p*-Value
Caregiver characteristics				
Parent’s age	30 [9]	30 [9]	30 [8]	0.915
Parent ethnicity				
Hispanic	7 (2)	4 (2)	3 (2)	0.711
Non-Hispanic	330 (98)	209 (98)	121 (98)	
Parent gender				
Female	346 (96)	217 (96)	129 (96)	0.831
Male	15 (4)	9 (4)	6 (4)	
Highest parent education completed				
≤High school	212 (61)	134 (63)	78 (59)	0.461
>High school	135 (39)	80 (37)	55 (41)	
Marital status				
Not married	155 (43)	95 (42)	60 (44)	0.623
Married or partnered	210 (57)	134 (58)	76 (56)	
Employment status				
Unemployed	194 (54)	120 (53)	74 (55)	0.751
Employed	167 (46)	106 (47)	61 (45)	
Household characteristics				
Child’s age	4 [1]	4 [1]	5 [1]	0.383
Number of children	2 [1]	2 [1]	2 [1]	0.860
Number of people (supported by income)	4 [2]	4 [2]	4 [2]	0.242
Yearly household income				
<$20,000	176 (49)	108 (48)	68 (50)	0.635
≥$20,000	185 (51)	118 (52)	67 (50)	
Participation in assistance program (in the past 12 months) ^5^				
SNAP/Food Stamps	270 (75)	156 (69)	114 (84)	0.001
WIC	253 (70)	167 (74)	86 (64)	0.041

^1^ Categorical variables were presented as *n* (%) and non-normal continuous variables were presented as medians and interquartile ranges (IQR). IQR represents the difference between the upper and lower quartiles (Q3−Q1). ^2^ Chi-square tests were conducted to determine differences between categorical variables and binary food security status. ^3^ Mann-Whitney U tests were used to determine differences between non-normal continuous variables and binary food security status. ^4^ Households with low and very low food security status were categorized as food insecure and those with marginal or high food security were classified as food secure [1]. ^5^ SNAP, Supplemental Nutrition Assistance Program; TANF, Temporary Assistance for Needy Families; WIC, The Special Supplemental Nutrition Program for Women, Infants, and Children.

**Table 2:** Food resource management (FRM) self-confidence and FRM behaviors of Head Start caregivers in the study sample by household food insecurity, (*n* = 365) ^1^.

	Responses	Total Sample (*n* = 365)	Food Secure (*n* = 229)	Food Insecure (*n* = 136)	*p*-Value
**FRM self-confidence *n* (%)**					
How confident are you that you can choose the best-priced form of fruits and vegetables (fresh, frozen or canned)?	Not very confident	17 (5)	9 (4)	8 (6)	0.046
	Somewhat confident	80 (22)	44 (19)	36 (27)	
	Moderately confident	135 (37)	80 (35)	55 (40)	
	Very confident	131 (36)	94 (42)	37 (27)	
How confident are you that you can buy healthy foods for your family on a budget?	Not very confident	28 (8)	12 (5)	16 (12)	<0.001
	Somewhat confident	85 (23)	44 (20)	41 (30)	
	Moderately confident	127 (35)	78(34)	49(37)	
	Very confident	122 (34)	94 (41)	28 (21)	
How confident are you that you can cook healthy foods for your family on a budget?	Not very confident	18 (5)	10 (4)	8 (6)	<0.001
	Somewhat confident	83 (23)	36 (16)	14 (34)	
	Moderately confident	126 (34)	79 (35)	47 (35)	
	Very confident	137 (38)	103 (45)	34 (25)	
How confident are you that you can make a shopping list and stick to it?	Not very confident	31 (8)	16 (7)	15 (11)	0.008
	Somewhat confident	86 (24)	50 (22)	36 (26)	
	Moderately confident	113 (31)	63 (28)	50 (37)	
	Very confident	134 (37)	99 (43)	35 (26)	
How confident are you that you can compare prices of similar foods to find the best value?	Not very confident	21 (6)	9 (4)	12 (9)	**0.015**
	Somewhat confident	70 (19)	40 (18)	30 (22)	
	Moderately confident	122 (33)	71 (31)	51 (37)	
	Very confident	151 (42)	108 (47)	43 (32)	
**FRM behaviors *n* (%)**					
How often do you compare prices before buying food?	Never	17 (5)	13 (6)	4 (3)	0.761
	Rarely	21 (6)	13 (6)	8 (6)	
	Sometimes	77 (21)	49 (21)	28 (21)	
	Usually	122 (33)	77 (34)	45 (33)	
	Always	127 (35)	76 (33)	51 (37)	
How often do you plan meals before shopping for groceries?	Never	13 (4)	8 (4)	5 (4)	0.812
	Rarely	30 (8)	18 (8)	12 (9)	
	Sometimes	112 (31)	71 (31)	41 (30)	
	Usually	131 (36)	78 (34)	53 (39)	
	Always	75 (21)	51 (23)	24 (18)	
How often do you use a shopping list when grocery shopping?	Never	14 (4)	7 (3)	7 (5)	**0.016**
	Rarely	25 (7)	15 (6)	10 (7)	
	Sometimes	70 (19)	45 (20)	25 (19)	
	Usually	99 (27)	50 (22)	49 (36)	
	Always	156 (43)	111 (49)	45 (33)	
How often do you check food on hand before making a shopping list? *	Never	7 (2)	3 (1)	4 (3)	0.349
	Rarely	15 (4)	12 (5)	3 (2)	
	Sometimes	55 (15)	33 (15)	22 (16)	
	Usually	117 (32)	69 (30)	48 (35)	
	Always	170 (47)	111 (49)	59 (44)	
How often do you use grocery store flyers to plan meals?	Never	67 (19)	42 (18)	25 (18)	0.922
	Rarely	63 (17)	38 (17)	25 (18)	
	Sometimes	121 (33)	74 (32)	47 (35)	
	Usually	56 (15)	38 (17)	18 (13)	
	Always	57 (16)	36 (16)	21 (16)	
How often do you identify foods on sale or use coupons to save money? *	Never	26 (7)	14 (6)	12 (9)	0.453
	Rarely	21 (6)	16 (7)	5 (4)	
	Sometimes	108 (30)	64 (28)	44 (32)	
	Usually	105 (29)	65 (29)	40 (29)	
	Always	104 (28)	69 (30)	35 (26)	

^1^ Chi-square test was conducted to determine differences between categorical variables and binary food security status. * For expected cell counts less than 5, *p*-value from Fisher’s exact test was reported.

**Table 3:** Financial situation, practices and difficulties of Head Start caregivers in the study sample and by household food insecurity (*n* = 365) ^1^.

	Responses	Total Sample (*n* = 365)	Food Secure (*n* = 229)	Food Insecure (*n* = 136)	*p*-Value
Which of the following best describes your family’s financial situation? * *n* (%)	Very comfortable & secure	31 (9)	27 (12)	4 (3)	<0.001
	Able to make ends meet without much difficulty	98 (28)	88 (40)	10 (7)	
	Occasionally have some difficulty making ends meet	121 (34)	69 (31)	52 (40)	
	Tough to make ends meet but keeping head above water	91 (26)	32 (14)	59 (45)	
	In over your head	13 (3)	7 (3)	6 (5)	
**Financial practices *n* (%)**					
How often do you review your bills for accuracy? *	Never	14 (4)	13 (6)	1 (1)	0.112
	Rarely	31 (8)	16 (7)	15 (11)	
	Sometimes	47 (13)	29 (13)	18 (13)	
	Usually	132 (36)	85 (37)	47 (35)	
	Always	140 (39)	85 (37)	55 (40)	
How often do you pay your bills on time? *	Never	5 (1)	3 (1)	2 (1)	<0.001
	Rarely	13 (4)	4 (2)	9 (7)	
	Sometimes	59 (16)	30 (13)	29 (21)	
	Usually	140 (38)	78 (34)	62 (46)	
	Always	148 (41)	114 (50)	34 (25)	
How often do you pay more than the “minimum payment due” on your credit card bills?	Never	109 (33)	66 (32)	43 (37)	0.001
	Rarely	46 (14)	19 (9)	27 (23)	
	Sometimes	70 (22)	47 (22)	23 (20)	
	Usually	46 (14)	35 (17)	11 (9)	
	Always	55 (17)	42 (20)	13 (11)	
**Financial difficulties *n* (%)**					
Has there been a time when you could not pay your mortgage or rent, electricity or gas utilities, or important medical expenses?	Yes	141 (39)	55 (24)	86 (63)	**<0.001**
Were you evicted from a home or apartment for not paying the rent or mortgage? *	Yes	6 (2)	2 (1)	4 (3)	0.201
Has there been a time when you could not pay the full amount of gas, oil, or electricity bills?	Yes	159 (44)	72 (32)	87 (64)	**<0.001**
Have you used a cash advance service on any of your credit cards? *	Yes	15 (4)	5 (2)	40 (7)	**0.013**
Have you used a payday loan or other high interest loan?	Yes	11 (3)	4 (2)	7 (5)	0.107

* For cells with counts less than 5 in the chi-square analysis, *p*-value from Fisher’s exact test was reported.

**Table 4:** Simple and multiple logistic regression analyses of food resource management (FRM) self-confidence, FRM behaviors, and financial practices of Head Start caregivers with household food insecurity (*n* = 365).

	Simple Logistic Regression	Multiple Logistic Regression ^1^
FRM self-confidence		
Low	1.0	1.0
High	0.50 (0.32, 0.77)	0.54 (0.33, 0.87)
*p*-value	*p* = 0.002	*p* = 0.012
FRM behaviors		
Low	1.0	-
High	0.98 (0.64, 1.5)	-
*p-value*	*p* = 0.913	
**Financial practices**		
Low	1.0	1.0
High	**0.52 (0.32, 0.85)**	0.77 (0.46, 1.3)
*p-value*	***p* = 0.010**	*p* = 0.338

^1^ The model was adjusted for socio-economic characteristics found to be significant correlates of household food insecurity, namely participation in any assistance program (in the past 12 months) including SNAP/Food Stamps or WIC.

## Supplementary Materials

The following are available online at https://www.mdpi.com/2072-6643/12/8/2304/s1. Table S1. Sensitivity analysis to evaluate the association between food resource management (FRM) self-confidence with household food insecurity adjusting for significant and non-significant sociodemographic variables as potential confounders in the logistic regression models, and using imputed and non-imputed income values. Table S2: Sensitivity analysis to evaluate the associations between food resource management (FRM) self-confidence, FRM behaviors, and financial practices of Head Start caregivers by household food insecurity using simple and multiple linear regression analyses.
