# Supplementary material for: Caregiver’s Self-Confidence in Food Resource Management Is Associated with Lower Risk of Household Food Insecurity among SNAP-Ed-Eligible Head Start Families
Source: Nutrients. 2020 Jul 31;12(8):2304. doi: 10.3390/nu12082304 (PMC7468708; doi:10.3390/nu12082304)
Supplement: Supplementary file 1 [file nutrients-12-02304-s001.pdf]

Supplementary

Table S1. Sensitivity analysis to evaluate the association between food resource management (FRM) self-confidence with household food insecurity adjusting for significant and non-significant sociodemographic variables as potential confounders in the logistic regression models, and using imputed and non-imputed income values.

	Simple logistic regression	Multiple logistic regression[1] (n=306)	Multiple logistic regression[2] (n=245)
FRM self-confidence			
Low	1.0	1.0	1.0
High	0.50(0.32,0.77)	0.59(0.36,0.97)	0.46(0.26,0.80)
**p-value**	**p=0.002**	**p=0.037**	**p=0.006**
FRM behaviors			
Low	1.0	-	-
High	0.98(0.64, 1.5)	-	-
**p-value**	p=0.913		
Financial practices			
Low	1.0	1.0	1.0
High	0.52(0.32,0.85)	0.72(0.42,1.23)	0.64(0.35,1.19)
**p-value**	p=0.010	p=0.230	p=0.163

[1]The model was adjusted for significant and non-significant demographic and socio-economic characteristics, including parents' age, income (**imputed**), employment and participation in any assistance program (in the past 12 months) including SNAP and WIC.

[2] The model was adjusted for significant and non-significant demographic and socio-economic characteristics including parents' age, income (**non-imputed**), employment and participation in any assistance program (in the past 12 months) including SNAP and WIC.

Table 2. Sensitivity analysis to evaluate the associations between food resource management (FRM) self-confidence, FRM behaviors, and financial practices of Head Start caregivers by household food insecurity using simple and multiple linear regression analyses.

	Simple linear regression	Multiple linear regression[1]	Multiple linear regression[2]
		Beta-coefficients (95% CI)	
FRM self-confidence score	-1.14(-1.87,-0.41)	-0.95(-1.69,-0.21)	-0.87(-1.63,-0.11)
**p-value**	**p=0.002**	**p=0.012**	**p=0.026**
FRM behaviors score	0.31(-0.41,1.04)	-	-
**p-value**	p=0.394	-	-
Financial practices score	-0.88(-1.63,-0.12)	-0.18(-0.96,0.60)	-0.32(-1.12,0.49)
**p-value**	**p=0.023**	p=0.651	p=0.441

[1]The model was adjusted for socio-economic characteristics found to be significant correlates of household food insecurity, namely participation in any assistance program (in the past 12 months) including SNAP/Food Stamps or WIC.

[2]The model was adjusted for significant and non-significant demographic and socio-economic characteristics, including parents' age, income (imputed), employment and participation in any assistance program (in the past 12 months) including SNAP and WIC.
